# Single genome amplification and molecular cloning of HIV-1 populations in acute HIV-1 infection: implications for studies on HIV-1 diversity and evolutionary rate

**DOI:** 10.1093/ve/veaf099

**Published:** 2025-12-18

**Authors:** Anthony Y Y Hsieh, Amin S Hassan, Jamirah Nazziwa, Lovisa Lindquist, Sara Karlson, Jonathan Hare, Anatoli Kamali, Etienne Karita, William Kilembe, Matt A Price, Per Björkman, Pontiano Kaleebu, Susan Allen, Eric Hunter, Jill Gilmour, Sarah L Rowland-Jones, Eduard J Sanders, Joakim Esbjörnsson

**Affiliations:** Centre for Immuno-Oncology, University of Oxford, Nuffield Department of Medicine, Old Road Campus Research Building, Roosevelt Drive, Oxford, OX3 7DQ, United Kingdom; KEMRI/Wellcome Trust Research Programme, Kilifi, Kenya; Department of Translational Medicine, Wallenberg Lab, plan 6, Inga Marie Nilssons gata 53, Lund University, 214 28 Malmö, Sweden; Institute for Human Development, Aga Khan University, P.O. Box 30270 - 00100, Nairobi, Kenya; Lund University Virus Centre, Wallenberg Lab, plan 6, Inga Marie Nilssons gata 53, Lund University, Sweden; Department of Translational Medicine, Wallenberg Lab, plan 6, Inga Marie Nilssons gata 53, Lund University, 214 28 Malmö, Sweden; Lund University Virus Centre, Wallenberg Lab, plan 6, Inga Marie Nilssons gata 53, Lund University, Sweden; Department of Translational Medicine, Wallenberg Lab, plan 6, Inga Marie Nilssons gata 53, Lund University, 214 28 Malmö, Sweden; Lund University Virus Centre, Wallenberg Lab, plan 6, Inga Marie Nilssons gata 53, Lund University, Sweden; Department of Translational Medicine, Wallenberg Lab, plan 6, Inga Marie Nilssons gata 53, Lund University, 214 28 Malmö, Sweden; Lund University Virus Centre, Wallenberg Lab, plan 6, Inga Marie Nilssons gata 53, Lund University, Sweden; IAVI New York, 125 Broad Street, 9th Floor, New York, NY 10004, United States; IAVI Human Immunology Laboratory, Imperial College, Chelsea and Westminster Hospital, London, SW10 9NH, United Kingdom; IAVI New York, 125 Broad Street, 9th Floor, New York, NY 10004, United States; IAVI Nairobi, PO Box 340 KNH 00202, Nairobi, Kenya; Center for Family Health Research, 57 KK 19 Avenue, Building 2, Kigali, Rwanda; Center for Family Health Research, B22/F737 Mwembelelo Road, Emmasdale, Lusaka, Zambia; IAVI New York, 125 Broad Street, 9th Floor, New York, NY 10004, United States; Center for Family Health Research, B22/F737 Mwembelelo Road, Emmasdale, Lusaka, Zambia; Department of Translational Medicine, Wallenberg Lab, plan 6, Inga Marie Nilssons gata 53, Lund University, 214 28 Malmö, Sweden; Lund University Virus Centre, Wallenberg Lab, plan 6, Inga Marie Nilssons gata 53, Lund University, Sweden; Department of infectious diseases, Skåne University Hospital, Ruth Lundskogs gata 3. Plan 1, 205 02 Malmö, Sweden; Uganda Research Unit, Medical Research Council/Uganda Virus Research Institute and London School of Hygiene and Tropical Medicine, Plot 51-59 Nakiwogo Road, PO Box 49, Entebbe, Uganda; Center for Family Health Research, B22/F737 Mwembelelo Road, Emmasdale, Lusaka, Zambia; UCSF Department of Epidemiology and Biostatistics, 550 16th Street, 2nd Floor, Global Health & Clinical Sciences Building, San Francisco, CA 94158-2549, United States; Department of Pathology & Laboratory Medicine, School of Medicine, Emory University, 1364 Clifton Road NE, Atlanta, GA 30322, United States; Center for Family Health Research, B22/F737 Mwembelelo Road, Emmasdale, Lusaka, Zambia; Department of infectious diseases, Skåne University Hospital, Ruth Lundskogs gata 3. Plan 1, 205 02 Malmö, Sweden; Department of Pathology & Laboratory Medicine, School of Medicine, Emory University, 1364 Clifton Road NE, Atlanta, GA 30322, United States; Department of Infectious Diseases, Infection and Immunity, Faculty of Medicine, Imperial College, Exhibition Road, South Kensington, London, SW7 2AZ, United Kingdom; Centre for Immuno-Oncology, University of Oxford, Nuffield Department of Medicine, Old Road Campus Research Building, Roosevelt Drive, Oxford, OX3 7DQ, United Kingdom; KEMRI/Wellcome Trust Research Programme, Kilifi, Kenya; Aurum Institute, 33 Wrench Rd, Isando, Rustenburg and Johannesburg, 1600, South Africa; Centre for Immuno-Oncology, University of Oxford, Nuffield Department of Medicine, Old Road Campus Research Building, Roosevelt Drive, Oxford, OX3 7DQ, United Kingdom; Department of Translational Medicine, Wallenberg Lab, plan 6, Inga Marie Nilssons gata 53, Lund University, 214 28 Malmö, Sweden; Lund University Virus Centre, Wallenberg Lab, plan 6, Inga Marie Nilssons gata 53, Lund University, Sweden

**Keywords:** HIV-1, single genome amplification, molecular cloning, acute HIV-1 infection, phylogenetics, HIV-1 diversity, HIV-1 evolution, transmitted founder virus

## Abstract

**Background:**

Human immunodeficiency virus type 1 (HIV-1) is one of the fastest-evolving human pathogens. Understanding HIV-1 transmission, within-host adaptation, and evolutionary dynamics is pivotal for development of interventions and vaccines. HIV-1 infection is generally caused by a single transmitted founder virus (TFV), and TFV sequences are typically obtained using single genome amplification (SGA). However, suboptimal sample quality can cause sequencing failures, representing considerable losses considering the scarcity of acute HIV-1 infection (AHI) samples. Sequencing failures may be mitigated by molecular cloning (MC), which can be less vulnerable to sample quality but more susceptible to polymerase chain reaction (PCR) errors. Here, we explore the feasibility of supplementing SGA with MC data using samples from clinical and research cohorts to determine whether sequence diversity and evolutionary rate estimates are comparable between the techniques.

**Methods:**

Plasma samples were selected from participants with documented AHI from an East African research cohort (the International AIDS Vaccine Initiative, 2006–2011) and a clinical cohort from Sweden (1983–2011). SGA and MC sequencing were done on the HIV-1 *env V1-V3* region (~940 base pairs). Within-host sequence diversity was determined from maximum likelihood phylogenetic trees, and evolutionary rate by Bayesian phylogenetic analysis. Highlighter plots, Hamming distances, and assessment of star phylogenies were used to quantify TFVs.

**Results:**

One hundred participants (median age 30.3 years, 15% female), contributing 350 samples from four longitudinal time points, 10–540 days post-infection, met the inclusion criteria. SGA succeeded on 90% of research cohort and 48% of clinical cohort samples. Comparative analysis of linked SGA and MC data from 10 samples indicated that approximately eight sequences were necessary for diversity estimates. Consistently higher sequence diversity was observed among MC relative to SGA sequences (median [IQR]: 0.009 [0.003, 0.015] and 0.004 [0.001, 0.012] substitutions/site, *P* = .002), whereas evolutionary rates were comparable between the two methods (0.016 [0.012, 0.019] and 0.011 [0.008, 0.020] substitutions/site/year, *P* = .232). Five participants with samples obtained within 45 days post-infection were eligible for TFV quantification, and all found to have one TFV using both techniques.

**Conclusion:**

MC data is a suitable supplement for SGA-based HIV-1 studies to preserve the value of precious samples for analysis of evolutionary rate, but not for sequence diversity.

## Introduction

Despite a heterogeneous human immunodeficiency virus type 1 (HIV-1) population seen in chronic infection, newly infected recipients mostly present with a genetically homogeneous virus population during acute HIV-1 infection (AHI) (Zhu et al. 1993, Delwart et al. 2002). This ‘bottleneck’ effect during HIV-1 transmission is typically a result of a single transmitted founder virus (TFV) infection (Learn et al. 2002, Derdeyn et al. 2004, Keele et al. 2008, Salazar-Gonzalez et al. 2008, 2009, Abrahams et al. 2009). Selection of the TFV is an elaborate confluence of route of infection, viral fitness, and host immunity (Abrahams et al. 2009, Carlson et al. 2014, Joseph et al. 2015). Following the establishment of infection, HIV-1 genetic diversity within the host accumulates due to genetic drift and selection during the chronic stage of infection (Shankarappa et al. 1999, Lemey et al. 2007, Esbjörnsson et al. 2012, Maldarelli et al. 2013). Over the past two decades, characterizing the evolution of HIV-1 into a highly heterogeneous population during the course of infection has become an important field of study with implications for virus control, vaccine design, and cure research (Simmonds et al. 1990, Derdeyn et al. 2004, Keele et al. 2008, Goonetilleke et al. 2009, Salazar-Gonzalez et al. 2009, Ho et al. 2013, Jones et al. 2020). Hence, the continual study of intra-host virus diversity in different populations and settings remains a priority to inform the development of a cure or effective vaccine towards HIV-1—particularly in AHI, wherein virus adaptation to a new host has yet to be fully characterized.

Early studies to characterize intra-host virus diversity used molecular cloning (MC) of PCR-amplified HIV-1 genes to isolate and determine individual HIV-1 sequences (Poss et al. 1995, Delwart et al. 2002, Learn et al. 2002, McCutchan et al. 2005). However, this method is susceptible to *Taq* polymerase errors, such as template switching, which may lead to cloning and sequencing of recombinant amplicons (Yang et al. 1996, Fang et al. 1998), thus overestimating diversity estimates and biasing evolutionary analysis. It is also possible that original template sequences are not proportionately amplified prior to MC, and thereby non-reflective of quasispecies composition (Liu et al. 1996), resulting in errors in diversity estimates.

Despite the popularity of next-generation sequencing approaches, the gold standard for measuring intra-host virus diversity is single-genome amplification (SGA), which involves isolating individual HIV-1 genomes through limiting dilution (Palmer et al. 2005, Salazar-Gonzalez et al. 2008). Importantly, selection of individual sequences by limiting dilution occurs before PCR amplification, and thus any potential PCR error would manifest within reads of a sequenced template. In contrast, PCR errors in MC accumulate prior to the selection of clones and would be dispersed across multiple sequenced templates. Therefore, SGA PCR errors can be identified and filtered out in the analysis pipeline, whereas MC PCR errors may be misattributed as diversity in the original sample.

Despite the superior accuracy of the SGA method, by amplifying the starting material prior to selection of clones, the MC method may be more likely to generate enough sequences for diversity and evolutionary rate analysis in circumstances where starting material is low and/or sample quality is suboptimal. Given that intra-host diversity studies are often characterized from comparatively scarce acute HIV-1 samples, instances in which SGA fails to generate sufficient sequences for analysis represent a non-trivial loss. Thus, for studies in which achieving adequate statistical power is hampered by the rarity of samples, it may be worthwhile to recover these sequences using MC. This is especially true given that many more single genomes would have to be attempted for amplification (a costly approach) to match the sensitivity of the MC method.

In this study, we explored the feasibility of supplementing SGA with MC data as part of a large research cohort from the International AIDS Vaccine Initiative (IAVI) and a historical clinical cohort from Sweden (Hassan et al. 2017, 2021, Nazziwa et al. 2024). We compared the estimated number of TFVs, HIV-1 sequence diversity, and HIV-1 evolutionary rate, as measured from sequences generated by these two techniques. We also assess the number of sequences necessary for diversity measurements for both methods. We hypothesized that sequence diversity would be higher when measured using MC compared to SGA due to errors inherent to the former methodology, but that the within-host evolutionary rate would be comparable between sequences generated by the two techniques. We also assessed the utility of qPCR quantification of HIV-1 genomes as an additional step in the SGA workflow, both to inform the limiting dilution and to use its concordance with clinically determined HIV-1 plasma viral load (pVL) as an indicator of sample quality.

## Methods

### Study participants

Study participants were selected based on our work described elsewhere (Hassan et al. 2017, 2021, Nazziwa et al. 2024). Briefly, participants were adults (≥18.0 years old) enrolled either in a research cohort (IAVI protocol C) (Kamali et al. 2015) 2006–11 from sites in Kilifi, Kenya; Kigali, Rwanda; Masaka, Uganda; and Lusaka, Zambia; or in a routine historical clinical cohort at the Skåne University Hospital Lund and Malmö, Sweden during 1983–2011. Eligibility included participants with AHI, as defined by samples collected at either Fiebig stage I (HIV-1 RNA positive) or Fiebig stage II (HIV-1 p24 antigen positive but with a negative HIV-1 antibody test) (Fiebig et al. 2003). Estimated date of infection (EDI) was defined as 10 days before the date of the first PCR-positive test (with a negative antibody or p24 antigen detection), or 14 days before the date of the first positive p24 antigen test (with a negative antibody test). Plasma samples from four longitudinally matched time points were obtained based on the number of days from the EDI as follows: Time point I (10–14 days); time point II (30 ± 15 days); time point III (90 ± 30 days); and time point IV (360 ± 180 days). Plasma samples from the research and clinical cohorts were stored at -80°C and -20°C, respectively. Participants enrolled in the research cohort provided written informed consent, and ethics approvals were obtained from ethics review boards of each participating country (Kamali et al. 2015). Approvals were obtained from ethics boards at each site, including the Kenya Medical research Institute science and ethics review unit, Rwanda National Ethics Committee, Uganda Virus Research Institute Science and Ethics Committee, Uganda National Council of Science and Technology, University of Zambia Research Ethics Committee, and Emory University Institutional Review Board (Kamali et al. 2015, Price et al. 2021). Ethics approval for the clinical cohort was obtained from the Lund University Ethical Review Board, Sweden (Dnr 2013/772).

### Single genome amplification and sequencing

SGA and sequencing was done as previously described (Esbjörnsson et al. 2010, 2011, 2012, Hassan et al. 2021). Briefly, archived plasma samples were retrieved, thawed, and 100 μl aliquots used for HIV-1 RNA extraction using the RNeasy lipid tissue Mini Kit (Cat# 74804, Qiagen) as per manufacturer’s instructions with minor modifications (Esbjörnsson et al. 2010). Electron microscopy-quantified HIV-1 virions (Cat# 10–118-000, Advanced Biotechnologies Inc) spiked in phosphate buffered saline (PBS) and neat PBS were used as positive and negative controls, respectively. A two-step RT-PCR protocol was used to amplify HIV-1 genomes. In the first step, the samples were reverse transcribed using random hexamers (Cat# N8080127, Thermo Fisher Scientific) to generate cDNA templates using the SuperScript™ IV Reverse Transcriptase Kit according to manufacturer’s instructions (Cat# 18090010, Thermo Fisher Scientific). Unlike conventional SGA workflows, a qPCR step was introduced. Specifically, the cDNA templates were quantified by qPCR using the SYBR™ Select Master Mix kit as per manufacturer’s instructions (Cat# 44–729-08, Applied Biosystems) using primers AP1_L1 (forward, 5′- GCCTCAATAAAGCTTGCCTTGA-3′) and AP1_L2 (reverse, 5′- GGCGCCACTGCTAGAGATTTT-3′). The qPCR results were used to inform calculations for serial limited dilutions of cDNA templates, aiming at 0.4 copies/μl (one copy of template in 2.5 μl) input for SGA. In the second step, diluted cDNA templates were used for outer and nested PCR using gene-specific primers targeting the HIV-1 *env V1-V3* region (approximately 940 base pairs, nucleotides 6430–7374 in HXB2; GenBank accession number K03455). Primers JE12F (forward) and V3A_R2 (reverse), primers E20A_F (forward) and JA169_R (reverse) were used for outer and nested PCR, respectively (Mild et al. 2007). All PCR reactions were done using the DreamTaq Green DNA Polymerase kit as per manufacturer’s instructions (Cat# EP0712, Thermo Fisher Scientific). Nested PCR products were visualized using agarose gel electrophoresis, and successful amplicons were retrieved, purified, and sequenced by the BigDye Terminator Cycle Sequencing Kit, using the primers E20A_F and JA169_R according to manufacturer’s instructions (Applied Biosystems). Twenty-three SGAs were targeted for sequencing for each sample.

### Molecular cloning and sequencing

MC and sequencing were done as previously described (Esbjörnsson et al. 2010). Briefly, the HIV-1 *V1-V3 env* region (as above) was amplified from 5 μl of the extracted HIV-1 RNA eluate that was used for SGA. Specifically, the outer primers used in the SGA approach (JE12F and V3A_R2) were used for one-step RT-PCR (SuperScript™ III One-Step RT-PCR System with Platinum® Taq DNA Polymerase, ThermoFisher Scientific); and the nested primers used above (E20A_F and JA169) were used for nested PCR (DreamTaq DNA Polymerase, ThermoFisher Scientific), as previously described (Leitner et al. 1996, Esbjörnsson et al. 2010). The amplified *V1-V3* region was then cloned using the TOPO™ TA Cloning™ Kit with One Shot™ TOP10 Chemically Competent *Escherichia coli* (ThermoFisher Scientific) according to the manufacturer’s instructions. Twenty-three colonies were routinely picked from each sample and amplified with DreamTaq DNA Polymerase (ThermoFisher Scientific) using conventional M13 primers (−20 and −24). Individual clones were purified (MinElute PCR Purification Kit, Qiagen) and sequenced by the BigDye Terminator Cycle Sequencing Kit (Applied Biosystems), using the primers E20A_F and JA169_R, according to the manufacturers’ instructions.

### Sequence data management

An automated workflow was set up in Geneious Prime Version 2023.1.2 Build 2023-04-27 14:16 (https://www.geneious.com) for sequence data management. Briefly, forward and reverse sequence reads were compiled, poor-quality ends trimmed, and *de novo* assembly done using default settings. Assembled contigs were mapped to the HXB2 reference sequence (GenBank accession number K03455), prior to the generation of a global alignment using the Clustal algorithm. The alignment was manually inspected prior to downstream analyses. A Neighbour Joining (NJ) phylogenetic tree (substitution model: Tamura-Nei, as per default settings) was explored for potential sample mix-up, sample mislabelling, and contamination. Sequences suggestive of a sample mix-up, mislabelling, or contamination were excluded from further analysis. The Pairwise Homoplasy Index (PHI) test was applied together with an in-house Perl script to iteratively and exhaustively screen for putative recombinants, as previously described (Bruen et al. 2006, Palm et al. 2019). For samples included in the diversity and evolutionary rate analyses (described below), the PHI test was complemented with a full exploratory recombination scan in RDP5 (Martin et al. 2021). Sequences suggestive of putative recombinants were excluded from further analysis. To assess the effects of recombinants, both complete alignments and alignments without putative recombinants were used in downstream analyses, respectively.

### Analysis of within-host diversity

To enable comparisons between SGA and MC, participants with at least one sample from which sequences generated using both SGA and MC were eligible. To assess the number of sequences needed for HIV-1 diversity estimation, the influence of the sequence abundance on the estimated diversity was explored. Participants with at least 15 SGA and MC sequences, respectively, were eligible. Two sequences were randomly selected from each sample. The sequence pairs from all samples were then aligned and used to construct an ML tree in IQ-TREE (settings: GTR + F + I) (Nguyen et al. 2015), from which the sample-wise diversity estimates were extracted using an in-house Perl script (Esbjörnsson et al. 2012). This was then iterated 100 times, resulting in 100 sample-specific diversity estimates based on two randomly selected sequences per sample. Next, these steps were independently repeated by adding one randomly selected sequence for each step until 15 sequences had been included for each sample, resulting in a total of 14 × 100 diversity estimates generated for each sample. The effects of the incremental increase in sampling were assessed by the longitudinal trend of the diversity IQR estimations.

The sample-wise diversity estimates were calculated by constructing maximum likelihood (ML) phylogenies in IQ-TREE (Nguyen et al. 2015). First, K3Pu + F + G4 was identified as the best-fitting model of sequence evolution, according to the lowest Bayesian Information Criterion (BIC) using the built-in feature ModelFinder (Kalyaanamoorthy et al. 2017). The G4 parameter was then substituted for the FreeRate parameter (R4), which recently has been suggested to better estimate branch lengths, for a comparison with the traditional gamma rate model (Fig. S1) (Ferretti *et al.* 2025). For each model, 1000 bootstrap phylogenies were reconstructed. The same Perl script as described above was then used to extract the within-host genetic diversity for each time point by averaging pairwise tree distances between sequences obtained from the same sample time point, as previously described (Esbjörnsson et al. 2012).

### Analysis of transmitted founder viruses

For each participant, sequences generated from either time point I or time point II (if time point I sequences were not available) were used for the determination of TFVs, as described (Keele et al. 2008). Briefly, all SGA and MC sequences were aligned in Geneious Prime using the Clustal algorithm with default settings, prior to extraction into separate alignments for each participant and time point. All alignments were manually inspected prior to downstream analyses. The number of TFVs was then determined using a three-pronged approach (HIV Sequence Database, National Institutes of Health, https://www.hiv.lanl.gov). First, the sequence alignments were visually inspected using the Highlighter tool (HIV Sequence Database, National Institutes of Health, https://www.hiv.lanl.gov/content/sequence/HIGHLIGHT/highlighter_top.html). Homogeneously distributed alignments were considered single TFV infections. Depending on the heterogeneity of the distribution of nucleotide substitution sites, alignments with two, three, or more distinguishable subpopulations were considered as two, three, or more TFV infections, respectively. Second, the sequence alignments were used to estimate pairwise Hamming distances (HDs), to explore their frequency distribution, mean of best fitting distribution, and goodness of fit *P*-values using the Poisson-Fitter tool (HIV Sequence Database, National Institutes of Health, https://www.hiv.lanl.gov/content/sequence/POISSON_FITTER/poisson_fitter.html). Graphical distribution of the HDs with one, two, three, or more peaks and with supporting statistics were considered single, two, three, or more TFV infections, respectively. Third, the sequence alignments were used to generate NJ phylogenies in Geneious Prime (substitution model: Tamura-Nei, as per default settings), which were further explored in an unrooted radial layout. Star-shaped phylogenies (with all sequences forming a single cluster, suggesting a monophyletic lineage) were considered single TFV infections. Bifurcated phylogenies (with sequences forming two, three, or more main clusters, suggesting a polyphyletic lineage) were considered as two, three, or more TFV infections, respectively. A consensus was determined based on majority across the three methods.

### Analysis of within-host evolution

The possibility of supplementing SGA data with MC data was explored by comparing the evolutionary rates of SGA samples only (SGA-exclusive) compared to when one sample per participant was exchanged for the corresponding sample generated using MC (MC-substituted). First, the temporal signal was assessed at participant level in TempEst v1.5.3 (Rambaut et al. 2016). ML trees were generated for each participant using IQ-TREE (Nguyen et al. 2015) (K3Pu + F + G4 or R4 according to the lowest BIC in ModelFinder (Kalyaanamoorthy et al. 2017) when analysing all sequences together). Again, the R4 parameter was included for a comparison between the gamma and FreeRate models (Fig. S1) (Ferretti *et al.* 2025). The Bayesian Evolution Analysis Utility (BEAUti) (Suchard et al. 2018) was used to set up .xml files for sequence analysis in Bayesian Evolutionary Analysis by Sampling Trees (BEAST) (Suchard et al. 2018). Since the objective was to compare MC-substituted and SGA-exclusive data, model priors were identical across all participants and sequencing methods. Nucleotide substitution rates were estimated using the Hasegawa-Kishino-Yano (HKY) substitution model with estimated base frequencies, four gamma categories, and two data partitions into codon positions (1 + 2, 3). Furthermore, a strict clock model with a constant coalescent population size was set as tree prior. The clock and tree models were unlinked for each participant, whereas the substitution and demographic models were linked across participants. The MC-substituted and SGA-exclusive analyses were run separately, and in duplicates for 100 million Markov Chain Monte Carlo (MCMC) iterations with sampling done after every 10 000 iterations, respectively. Log files were combined in Logcombiner (Suchard et al. 2018) and analysed in Tracer (Suchard et al. 2018) with a burn-in period of 10%. Effective Sample Sizes (ESSs) > 100 reflected sufficient posterior distributions of model parameters.

### Statistical analysis

Participant demographic data were presented with summary statistics. Continuous data were presented using medians and interquartile ranges (IQRs), while categorical data were presented using frequencies and percentages. Chi-squared and Mann–Whitney *U* tests were done to compare sample quality between the research and clinical cohorts. Pearson’s correlations were used to characterize the concordance between the quantity of HIV-1 plasma VL, as historically determined from the research site/clinic, and during the current sequencing from the HIV-1 qPCR step introduced in this study. Cochran–Armitage test for trend was used to compare recombinant sequences with time point. Absolute differences in diversity and evolutionary rate between MC and SGA data were assessed using a two-sample Wilcoxon signed-rank test of medians. *P*-values below .05 were considered statistically significant.

## Results

### Characteristics of study participants

Overall, 100 participants were eligible for SGA sequencing (median age, 30.3 [IQR, 24.3–35.9] years and male [*n* = 85, 85%], Table 1). Of these, 74 participants were from the research cohort and 26 participants from the clinical cohort. Overall, 50 of the targeted samples were missing from 39 participants, resulting in 350 available samples in total (Fig. S2). All participants had at least one sample available.

**Table 1:** Characteristics of participants with AHI from the clinical and research cohorts in this study

**Baseline variables**		**Clinical cohort (*n* = 26)**	**Research cohort (*n* = 74)**	**Overall (*n* = 100)**
**Age (years)**	Median (IQR)	35.3 (28.2–40.2)	29.3 (23–33.6)	30.3 (24.3–35.9)
**Age group (years)**	18.0–24.9	5 (19.2)	22 (29.7)	27 (27.0)
	25.0–34.9	8 (30.8)	38 (51.4)	46 (46.0)
	35.0+	13 (50.0)	14 (18.9)	27 (27.0)
**Sex**	Female	0 (0.0)	15 (20.3)	15 (15.0)
	Male	26 (100)	59 (79.7)	85 (85.0)
**Year of infection**	<2009	16 (61.5)	26 (35.1)	42 (42.0)
	2009–10	3 (11.5)	38 (51.4)	41 (41.0)
	2011+	7 (26.9)	10 (13.5)	17 (17.0)
**Risk group**	HET	5 (19.2)	46 (62.2)	51 (51.0)
	MSM	21 (80.8)	28 (37.8)	49 (49.0)
**Country**	Rwanda	0 (0.0)	14 (18.9)	14 (14.0)
	Uganda	0 (0.0)	13 (17.6)	13 (13.0)
	Kenya	0 (0.0)	32 (43.2)	32 (32.0)
	Zambia	0 (0.0)	15 (20.3)	15 (15.0)
	Sweden	26 (100)	0 (0.0)	26 (26.0)
**HIV-1 subtype**	A1	1 (3.8)	46 (62.2)	47 (47.0)
	B	8 (30.8)	0 (0.0)	8 (8.0)
	C	1 (3.8)	18 (24.3)	19 (19.0)
	D	0 (0.0)	6 (8.1)	6 (6.0)
	Others^a^	4 (15.4)	2 (2.7)	6 (6.0)
	Missing	12 (46.2)	2 (2.7)	14 (14.0)
**TFVs**	Single	10 (71.4)	54 (78.3)	64 (77.1)
	Multiple	4 (28.6)	15 (21.7)	19 (22.9)
	Missing	17 (20.5)	5 (7.2)	17 (20.5)

a Others: HIV-1 subtypes F1 (*n* = 1), G (*n* = 1), and recombinants A2D (*n* = 1), AE (*n* = 2), and BG (*n* = 1). Research cohort recruited from Kenya, Rwanda, Uganda, and Zambia, and clinical cohort recruited from Sweden. Abbreviations: IQR (interquartile range), HET (heterosexual), and MSM (men who have sex with men).

### qPCR measurements for limiting dilution and sample quality estimates

SGA relies on limiting dilution to isolate HIV-1 single genomes, and the dilution itself was informed by quantifying the cDNA using qPCR. The qPCR data were also used to evaluate sample quality by comparing with the visit-specific HIV-1 pVL. HIV-1 pVL and qPCR data were available for 282 longitudinal visits from 92 participants. Of these, the correlation between HIV-1 pVL and qPCR was R^2^ = 0.44 (*P* < .001) for research samples and R^2^ = 0.29 (*P* < .001) for clinical samples (Fig. S3). Among the 277 visits with detectable HIV-1 pVL, 57 (21%) could not be detected by qPCR (Fig. S4). This was higher in the clinical cohort than in the research cohort (27 [66%] versus 30 [13%] samples, *P* < .001), and was associated with older sample collection date (median difference 535 days, *P* = .008). In sensitivity analyses excluding samples collected before 2005, the effect of sample age could not be detected in either clinical (*P* = .14) or research (*P* = .15) cohorts. When the analysis was restricted to those with detectable HIV-1 qPCR, the correlation with historic HIV-1 pVL improved to R^2^ = 0.62 (*P* < .001) and R^2^ = 0.48 (*P* = .006) for the research and clinical study participants, respectively (Fig. S3).

### Single genome amplification and molecular cloning

**Figure 1:**
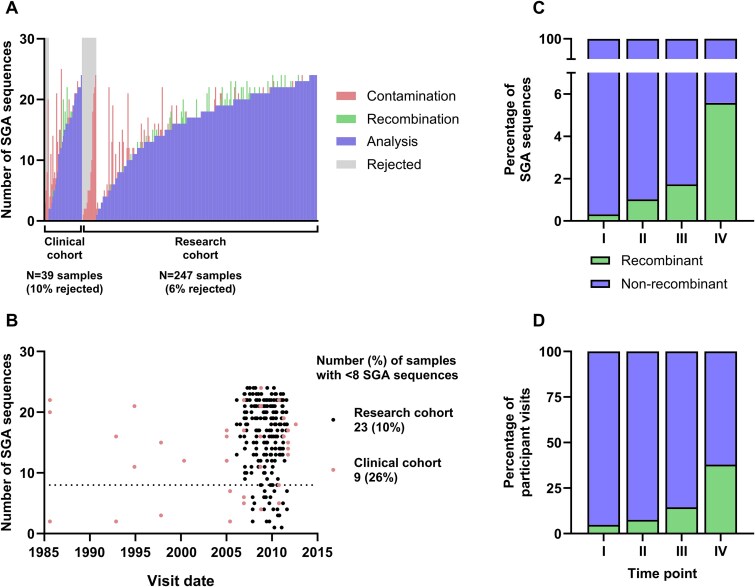
Summary of SGA sequences: (A) Numbers of SGA sequences that were analysed (blue), identified as recombinant (green), or rejected (grey) due to contamination (red) are shown, clinical and research cohorts were grouped separately, rejected sequences were arranged by ascending number of contaminated sequences, and accepted sequences were sorted by ascending number of analysed sequences; (B) relationship between visit date and number of sequences obtained per sample in research (black dots) and clinical (red dots) samples; (C) percentage of recombinant sequences among all obtained sequences, and (D) percentage of samples with recombinant sequences at each time point.

From the 350 available samples, 50 samples from 29 participants could not be PCR-amplified and, therefore, not sequenced. Of the remaining 300 samples successfully amplified and sequenced, 14 samples from 13 participants yielded no sequence data (Fig. S2). Among available samples, PCR amplification or sequencing failures were 52% in the clinical cohort and 10% in the research cohort. In total, 286 samples from 92 participants yielded sequencing data; however, 18 (6%) samples from 15 participants were excluded from further analysis because of potential contamination (Fig. 1). Finally, sequence data from 268 (94%) samples from 86 participants were included in downstream analyses (Fig. S2). Across all plasma samples, the mean ± standard deviation (SD) number of sequenced clones was 16.1 ± 5.9. The number of sequenced clones obtained per sample was higher in the research cohort compared to the clinical cohort (16.5 ± 5.7 sequences versus 13.5 ± 6.7 sequences, *P* = .005). There was no relationship between sample collection date and number of sequences obtained per sample (*P* = .17).

There were a total of 4391 SGA sequences. Of these, putative PCR-induced recombination was detected in 94 (2.1%) sequences. The proportion of putative recombinant sequences per total number of sequences increased with later time points, ranging from 0.3% in time point I to 5.6% in time point IV (*P* = .011, Fig. 1). When grouped by sample, the proportion of samples containing a putative recombinant sequence followed a similar pattern, in which 4.7% of time point I samples had recombinant sequences and 37.8% of time point IV participants had recombinant sequences (*P* < .001, Fig. 1).

### Assessment of the number of sequences needed for HIV-1 diversity estimation

**Figure 2:**
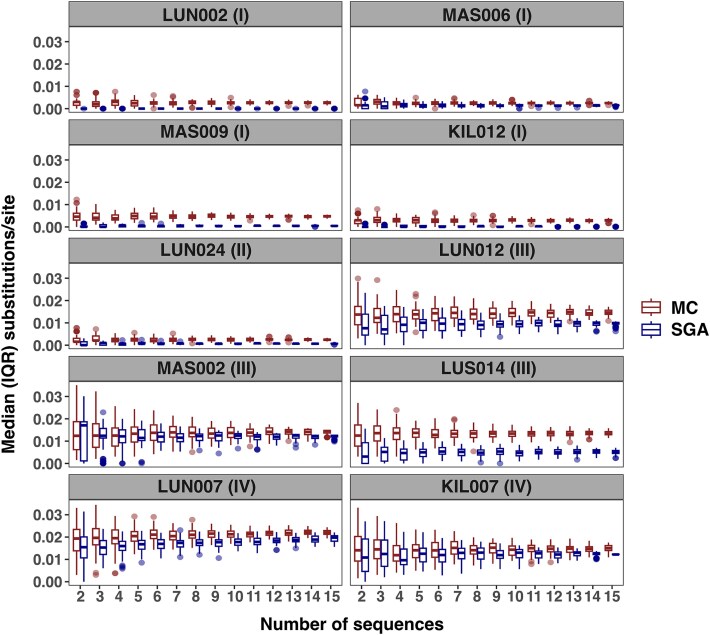
Assessment of the number of sequences needed for HIV-1 diversity estimation. Among participants with at least fifteen sequences (*N* = 10), two to fifteen sequences were randomly selected per participant over 100 iterations, respectively. The boxplots represent the median (IQR, range) estimates when sampling the number of sequences on the x-axis. MC (red) and single-genome amplification (blue). Panel headers indicate the anonymised participant identifier and time point sample used. Abbreviations: MC (molecular cloning), SGA (single genome amplification), IQR (interquartile range).

Ten samples comprised at least 15 MC and SGA sequences, respectively. When randomly sampling 2–15 sequences from the sequence pool 100 times to estimate the average pairwise distance after each round, the median diversity was relatively unaffected by the number of sampled sequences, with a median (IQR) difference between the highest and lowest median values per participant of 0.001 (0.001, 0.003) substitutions/site (Fig. 2). However, the IQRs were generally largest when two sequences were used and progressively decreased as more sequences were included. This effect was most pronounced in samples from chronic infection (samples III and IV), for which the difference between the largest and smallest IQRs per participant was 0.008 (0.008, 0.011). Here, a clear reduction of the IQR was observed when sampling up to eight to ten sequences, with negligible improvements thereafter (Fig. 2). In contrast, this effect was less pronounced for samples in AHI (time points I and II), for which the median (IQR) difference between largest and smallest IQRs per participant was 0.001 (0.001, 0.002).

Overall, 23 (10%) of the research cohort samples and 9 (26%) of the routine clinical cohort samples contained fewer than eight sequences and were not considered for comparison of diversity of SGA and MC (Fig. 1). Among the ten participants eligible for downstream analyses, a total of six (3.2%) and seven (3.3%) putatively recombinant sequences were detected in the SGA and MC samples, respectively. The SGA backbone used in the evolutionary analyses contained four (1.1%) putatively recombinant sequences. No additional recombinant sequences were detected by RDP5 compared to what had already been detected by the initial screen by the iterative PHI test.

### Comparison of diversity between SGA and MC

**Figure 3:**
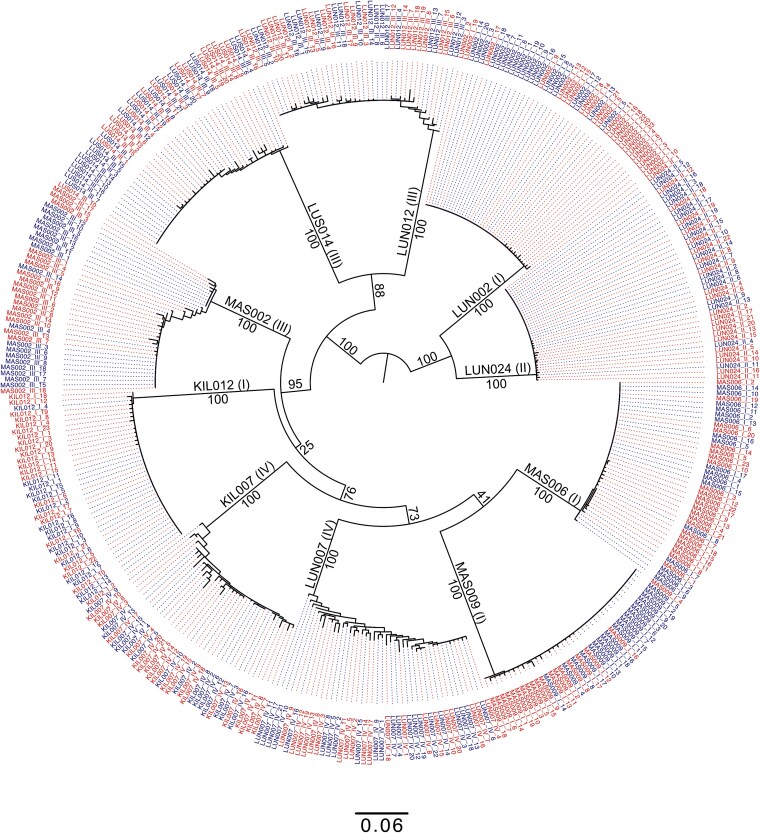
A phylogenetic tree showing relatedness of HIV-1 *env V1-V3* sequences generated from MC (red) and SGA (blue). Labels “LUN002 (I),” “LUN007 (IV),” “LUN012 (III),” and “LUN024 (II)” indicate samples from the clinical cohort, and the remaining samples are from the research cohort. Values in parentheses indicate visit number. The scale bar represents substitutions/site, and numbers on the branches indicate branch support as determined by the ultrafast bootstrap feature in IQ-TREE.

To compare measurements of diversity between SGA and MC methods, 10 participants were selected to ensure an even distribution across study sites, time points, and SGA sequences (Table 2). SGA yielded a mean ± SD of 18.6 ± 2.2 sequences for analysis, compared to 20.9 ± 1.8 sequences from MC. Moreover, *prima facie* data of the NJ phylogeny indicated that MC and SGA sequences from the same participant and time point clustered together, time point I sequences had more homogeneous sequences with shorter branch lengths compared to those from later time points (Fig. 3).

**Table 2:** Samples involved in the comparison between SGA and MC

			**MC**		**SGA**	
**Participant ID**	**Time point**	**Cohort**	**Number of sequences**	**TFVs**	**Number of sequences**	**TFVs**
LUN002	I	Clinical	23	Single	22	Single
LUN007	IV	Clinical	18	NA	16	NA
LUN012	III	Clinical	20	NA	15	NA
LUN024	II	Clinical	22	Single	19	Single
MAS002	III	Research	16	NA	17	NA
MAS006	I	Research	23	Single	17	Single
MAS009	I	Research	19	Single	20	Single
KIL007	IV	Research	19	NA	15	NA
KIL012	I	Research	23	Single	16	Single
LUS014	III	Research	19	NA	20	NA

Research cohort recruited from Kenya, Rwanda, Uganda, and Zambia, and clinical cohort recruited from Sweden. TFVs not investigated for time points III and IV. Abbreviations: ID (identifier) and NA (not applicable).

**Figure 4:**
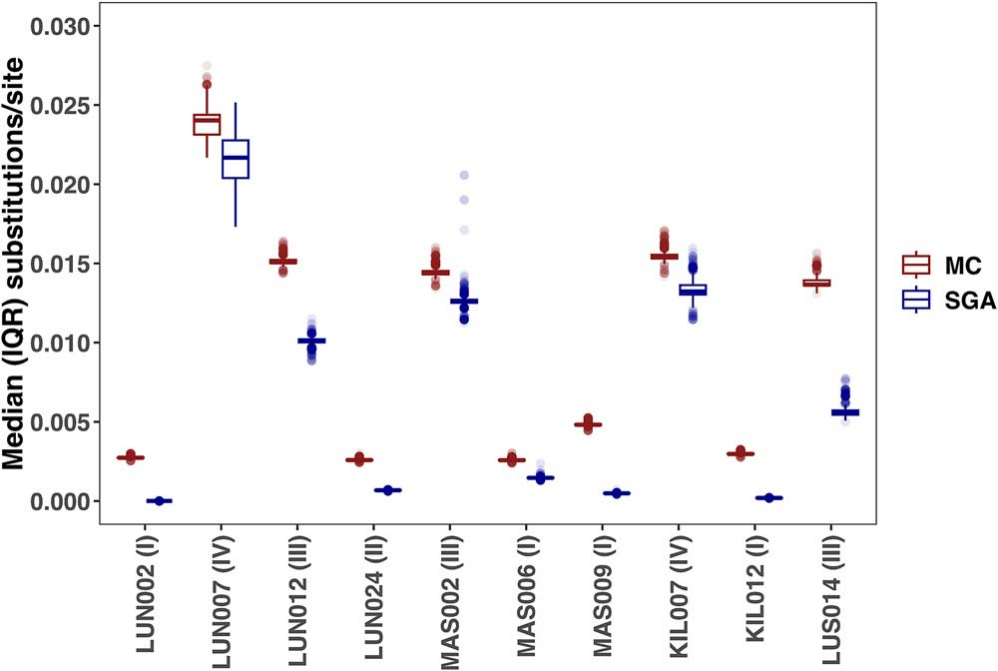
HIV-1 *env V1-V3* diversity compared between MC (red) and SGA (blue) methods. Participants from the clinical (LUN) and research (MAS, KIL, LUS) cohorts are shown. Values in parentheses indicate visit number. Abbreviations: MC (molecular cloning), SGA (single genome amplification), IQR (interquartile range).

The diversity analysis indicated consistently higher HIV-1 sequence diversity among the MC sequences compared with the corresponding SGA sequences (Fig. 4). The median (IQR) diversities between MC and SGA sequence data were 0.009 (0.003, 0.015) and 0.004 (0.001, 0.012) nucleotide substitutions/site, respectively (*P* = .002). The same pattern was observed when putatively recombinant sequences were included (0.010 [0.003, 0.017] and 0.004 [0.001, 0.012], *P* = .002, Fig. S5). Furthermore, the pattern of higher diversity among MC relative to SGA sequences was observed among participants from both the research and the clinical cohorts (Fig. 4). Finally, among participants selected for SGA and MC comparisons, five samples were either time point I or II, and thus eligible for TFV quantification (Table 2). All five samples were found to have a single TFV regardless of SGA or MC method used (Figs S6, S7 and S8).

### Comparison of evolutionary rates between SGA and MC

**Figure 5:**
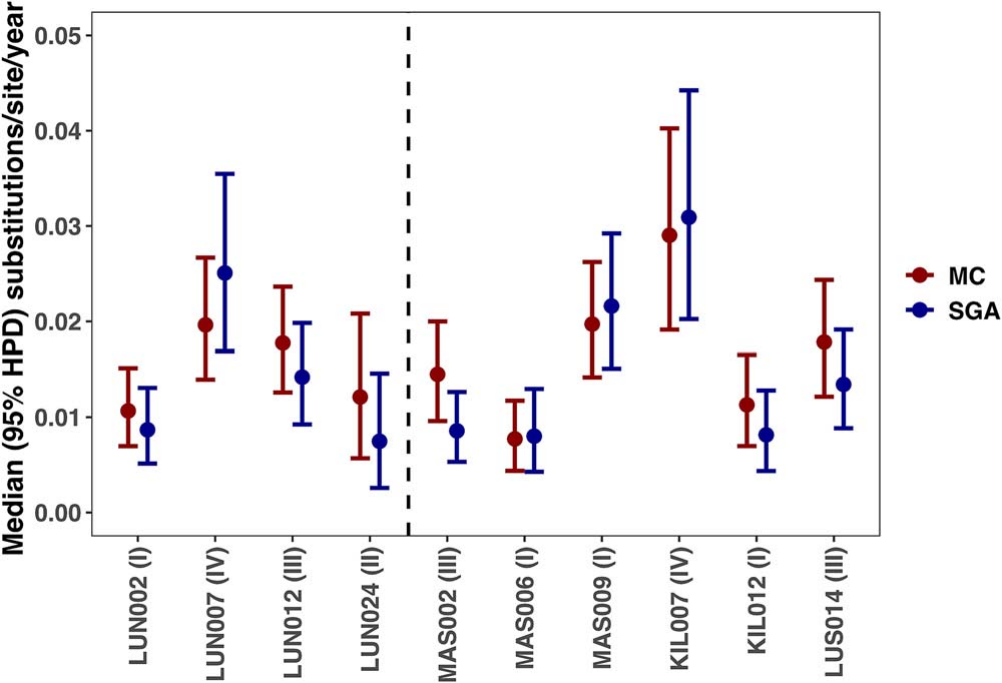
HIV-1 *env V1-V3* evolutionary rates compared between MC (red) and SGA (blue) methods. SGA estimates were generated using all available SGA samples. MC estimates were generated by replacing the sample in parentheses in the SGA alignment with the corresponding MC sample. Dotted line separates clinical (left) from research (right) cohort participants. Abbreviations: MC (molecular cloning), SGA (single genome amplification), HPD (highest posterior density).

Non-zero evolutionary rates were detected in all samples (Fig. 5). Furthermore, there was considerable between-patient variability in the HIV-1 evolutionary rates, regardless of whether the estimates were determined using SGA or MC methods. However, within-patient HIV-1 evolutionary rate estimates between SGA and MC methods were comparable with overlapping 95% highest posterior density intervals (median [IQR]: 0.016, [0.012, 0.019] and 0.011 [0.008, 0.020] substitutions/site/year, *P* = .232). The same pattern was observed when including putatively recombinant sequences (0.017 [0.011, 0.019] and 0.011 [0.008, 0.019], *P* = .193, Fig. S8). In addition, the within-host tree height estimates were comparable between the MC and SGA analyses (Table S1). There were no clear patterns in evolutionary rates based on the HIV-1 subtypes (Table S2).

## Discussion

Here, we present a comparison between SGA and MC methods to measure HIV-1 sequence diversity and evolutionary rate in participants with AHI. We corroborate previous findings by demonstrating that measured HIV-1 sequence diversity is higher by MC relative to SGA (Salazar-Gonzalez et al. 2008). We also show for the first time that measurements of within-host evolutionary rates are comparable between the two methods, suggesting that errors intrinsic to the MC method do not propagate into evolutionary rate. The analyses were done using samples from two sources with differing sample quality, and the findings were independent of sample quality and cohort type. Hence, for circumstances in which samples cannot be processed by SGA due to unavoidable variability in sample quality, the use of MC can be justified to supplement measurements of evolutionary rate.

Several studies have directly compared SGA and MC methods. Salazar-Gonzalez *et al.* reported higher diversity and prevalence of recombinant sequences by MC compared to SGA of HIV-1 *env* sequences within two participants (Salazar-Gonzalez et al. 2008). In a later study, Jordan *et al.* compared the two techniques in 17 participants to investigate inter-individual diversity in HIV-1 *pro-pol* sequences and found no differences in diversity (Jordan et al. 2010). However, the authors noted that a sufficient number of analysed sequences is necessary to detect low-prevalence quasispecies (Eric et al. 2024), and the necessary number of sequences of comparable sequence lengths to detect differences between the techniques is likely greater using *pro-pol* than *env*, given that the latter is less conserved and harbours highly variable regions. We used our dataset to explicitly show the necessary number of sequences for stable diversity estimates and observed eight sequences to be the approximate threshold. However, this is based on the within-host diversity of the population sampled in this study. It is possible that different thresholds exist for different populations, such as a chronic infection setting, in which diversity is likely to be higher.

Contending with poorer sample quality often accompanies studies using historical cohorts. In our analysis, research cohort samples returned more sequences, had fewer rejected sequences, and better concordance between qPCR measurement and HIV-1 pVL compared to clinical cohort samples. We observed that for several clinical cohort samples, historical HIV-1 pVL at visit could not be detected by qPCR when assayed years later. Although the clinical cohort tended to have older samples, we could not detect a relationship between sample age and quality in sensitivity analyses restricted to newer samples. It is possible that storage conditions (-80°C for research cohort and -20°C for clinical cohort samples) or cohort effects, such as differences in sample handling/processing, also played a role in sample degradation. Despite this, we show that the variability in diversity estimates between SGA and MC methods outweighs the variability between cohorts, suggesting that the heterogeneity in the samples used did not strongly affect the comparison.

Several studies have incorporated qPCR or digital PCR quantification of extracted HIV-1 genomes in the SGA workflow to inform the dilution to a single genome per sequencing reaction (Palmer et al. 2005, Lee et al. 2019). We expand on the utility of the qPCR data by describing the agreement between qPCR data and HIV-1 pVL at visit as a proxy of sample quality. We demonstrate that suboptimal sample quality in older samples is more likely to be reflected in divergence between HIV-1 qPCR and HIV-1 pVL concentrations. Thus, for studies in which variable sample quality is likely to lead to failed SGA sequencing, qPCR and HIV-1 pVL discordance could be used to screen for samples that would benefit from MC supplementation. Conversely, for studies in which sample quality is consistently high, the variability in HIV-1 pVL measurements is largely captured by qPCR. This suggests that the former may be used to inform SGA serial dilutions, bypassing the qPCR step. However, the performance and reliability of this warrant an empiric evaluation.

A variety of next-generation sequencing platforms have become commonplace in HIV-1 genomics research (Liang et al. 2011, Kijak et al. 2019), including studies of acute infection (Liang et al. 2011). While useful to achieve greater read depth and broader coverage of the HIV-1 genome, next-generation sequencing techniques often have limited read length and are vulnerable to sequencing errors and sample contamination compared to sequencing protocols based on Sanger sequencing (Brumme and Poon 2017). They also rely on *in silico* methods to stitch together sequencing fragments to form the original genomes. These limitations may constrain the usefulness of next-generation techniques in diversity/evolution studies. Large fragment single molecule sequencing could, however, be a uniquely viable substitute for SGA, and has been used to study full-length *env* sequences (Laird Smith et al. 2016). Although it is applicable for HIV-1 diversity/evolution studies, it remains vulnerable to recombination error, and robustness with low starting material has yet to be determined.

In our sequence analysis pipeline, we identify and exclude putative recombinant sequences from downstream analysis. Given the propensity of the MC method to introduce recombination errors (Yang et al. 1996, Fang et al. 1998), it is critical to delineate *in vivo* from PCR-induced recombination. However, our data suggest that this does not explain the entirety of the error intrinsic to the MC method, as even among samples that have passed screening for putative recombinants, estimated diversity was higher than the SGA data. Indeed, after screening for recombinant sequences, two samples in our analysis had no sequence diversity when measured by SGA, but several nucleotide substitutions were detected when measured by MC.

Our analysis benefits from a range of sample quality characteristics of a large, multi-site AHI cohort study. We directly compare SGA and MC methods on the same samples to truly describe how MC measurements of evolutionary rate can supplement SGA data in this context. The low number of SGA reads in several samples was realistic, and we show that although increasing the number of sequences did not affect diversity estimates, sampling sequences fewer than a threshold of approximately eight sequences often underestimated diversity estimates. This threshold did not appear to be different between SGA and MC data, with the overestimation of diversity using MC seeming to be present across the range of sequences used, even down to two sequences. This is likely because the error is introduced in the RT-PCR stage and is already present prior to clone selection and sequencing. This suggests that error inherent to the MC method is constant and not dependent on the number of sequences generated.

The value of human cohort studies that span a long period of time and/or across different settings is indisputable, but can often be associated with unavoidable variability in sample quality. This work is an effort to strike a balance between maintaining the quality of data and conserving the value of precious samples.

## Supplementary Material

Hsieh_SI_veaf099

## Data Availability

The datasets used and analysed in this study are available from the corresponding author on reasonable request.
