# Supplementary material for: Single genome amplification and molecular cloning of HIV-1 populations in acute HIV-1 infection: implications for studies on HIV-1 diversity and evolutionary rate
Source: Virus Evol. 2025 Dec 18;12(1):veaf099. doi: 10.1093/ve/veaf099 (PMC12815262; doi:10.1093/ve/veaf099)
Supplement: Hsieh_SI_veaf099 [file hsieh_si_veaf099.docx]

**SINGLE GENOME AMPLIFICATION AND MOLECULAR CLONING OF HIV-1 POPULATIONS IN ACUTE hiV-1 INFECTION: IMPLICATIONS FOR STUDIES ON HIV-1 DIVERSITY AND EVOLUTIONary rate**

**Authors:** Anthony Y.Y. Hsieh (1)*, Amin S. Hassan (2, 3, 4, 5)*, Jamirah Nazziwa (3, 5), Lovisa Lindquist (3, 5), Sara Karlson (3, 5), Jonathan Hare (6, 7), Anatoli Kamali (6, 8), Etienne Karita (9), William Kilembe (10), Matt A. Price (6, 10), Per Björkman (3, 5, 12), Pontiano Kaleebu (13), Susan Allen (10, 11, 14), Eric Hunter (10, 11, 14), Jill Gilmour (15), Sarah L. Rowland-Jones (1), Eduard J. Sanders (2, 16), Joakim Esbjörnsson (1, 3, 5)§

*Authors with equal contribution

**Author** **Affiliations:**(1) Centre for Immuno-Oncology, University of Oxford, UK; (2) KEMRI/Wellcome Trust Research Programme, Kilifi, Kenya; (3) Department of Translational Medicine, Lund University, Sweden; (4) Institute for Human Development, Aga Khan University, Nairobi, Kenya; (5) Lund University Virus Centre, Lund University, Sweden; (6) IAVI New York, USA; (7) IAVI Human Immunology Laboratory, Imperial College, London, United Kingdom; (8) IAVI Nairobi, Kenya; (9) Center for Family Health Research, Kigali, Rwanda; (10) Center for Family Health Research, Lusaka, Zambia; (11) UCSF Department of Epidemiology and Biostatistics, San Francisco, California, USA; (12) Department of infectious diseases, Skåne University Hospital, Malmö, Sweden; (13) Uganda Research Unit, Medical Research Council/Uganda Virus Research Institute and London School of Hygiene and Tropical Medicine, Entebbe, Uganda; (14) Department of Pathology & Laboratory Medicine, School of Medicine, Emory University, Atlanta, GA, USA; (15) Department of Infectious Diseases, Infection and Immunity, Faculty of Medicine, Imperial College, London, UK; (16) Aurum Institute, Rustenburg and Johannesburg, South Africa

**§Corresponding Author:**

Joakim Esbjörnsson

Department of Translational Medicine

Wallenberg Lab, plan 6, Inga Marie Nilssons gata 53

Lund University

214 28 Malmö, Sweden

Email: [Joakim.esbjornsson@med.lu.se](mailto:Joakim.esbjornson@med.lu.se)

**SUPPLEMENTARY MATERIAL**

**Figure S1. Diversity and phylogenetic signal depending on the sequencing method (MC/SGA), handling of recombinants (inclusion/exclusion), and the model of rate heterogeneity (Gamma/FreeRate).** The average pairwise diversity (A) and temporal signal (B) were estimated from maximum-likelihood phylogenetic trees, generated using the K3Pu+F model of sequence evolution, and either a gamma (G4) or FreeRate (R4) distribution for rate heterogeneity. Recombinant sequences were detected using the PHI test and RDP5, and then either retained or removed from the alignments. The diversity was calculated using an in-house Perl script, and the correlation was determined using TempEst. Two participants, LUN012 and KIL007, displayed discordant results for the temporal signal. To determine whether the stochastic tree-construction algorithm affected the results for these participants, the phylogenies were reconstructed ten times. For LUN012, this indicated that parallel evolution of multiple infecting strains resulted in two sublineages of the phylogeny, whose relative direction when assembled affected the signal. This was observed both with and without recombinants and using both the Gamma and FreeRate models (data not shown). For KIL007, for which there were variations in signal for both SGA and MC, with and without recombinants, there were no clear differences in signal across the ten replicates. There was a tendency towards smaller residuals with the Gamma model compared with the FreeRate model when the recombinants were included and SGA data was supplemented with MC data (panel C). A similar pattern was also seen for the SGA data without recombinants (panel C). X-axes in panel C represent time in days. Abbreviations: ID (identifier), MC (molecular cloning), SGA (single genome amplification), PHI (pairwise homoplasy index), RDP (recombination detection program).

**
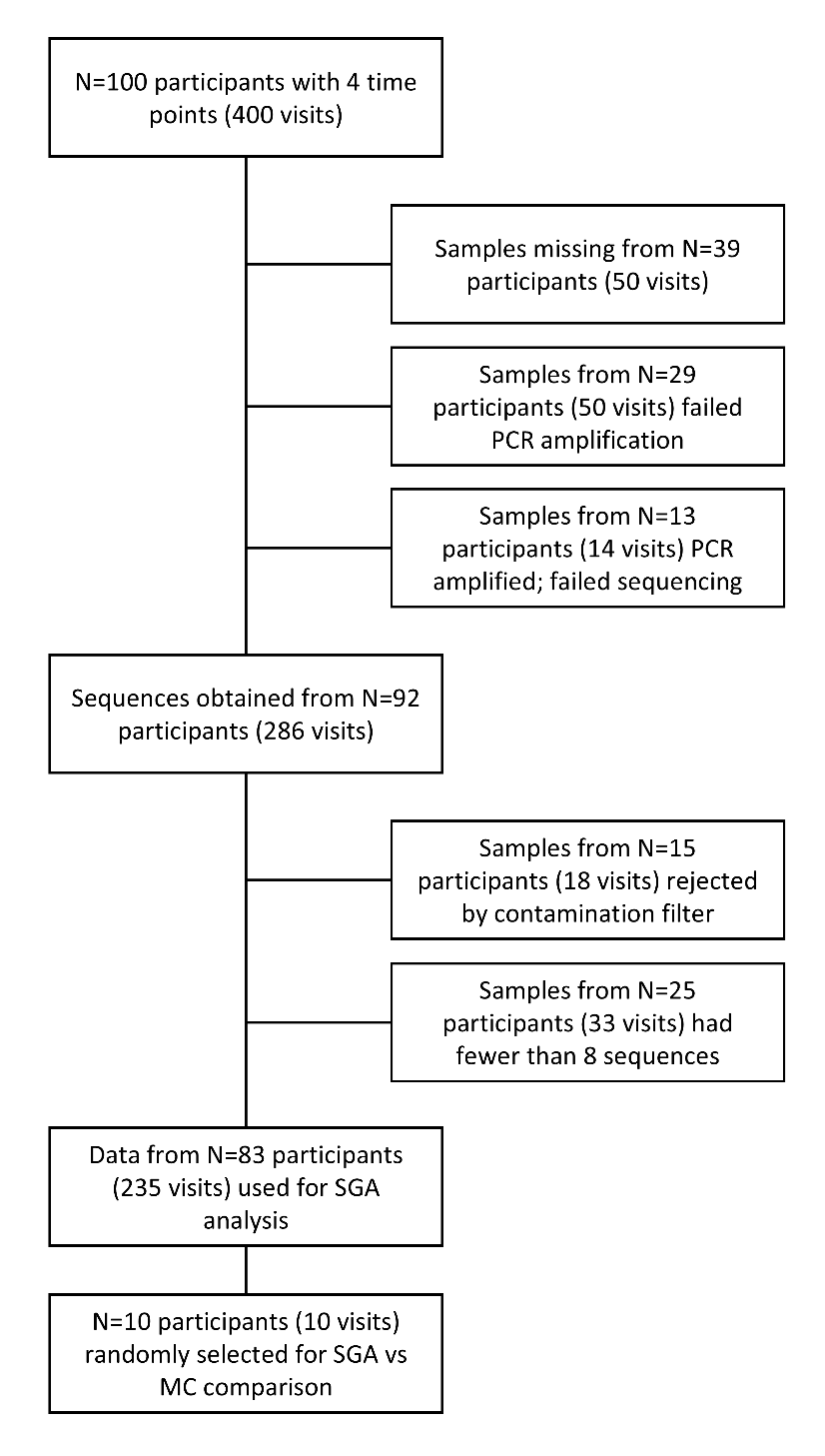
**

**Figure S2. Inclusion criteria of participants and samples for single genome amplification sequencing and downstream analysis.** Abbreviations: SGA (single genome amplification).


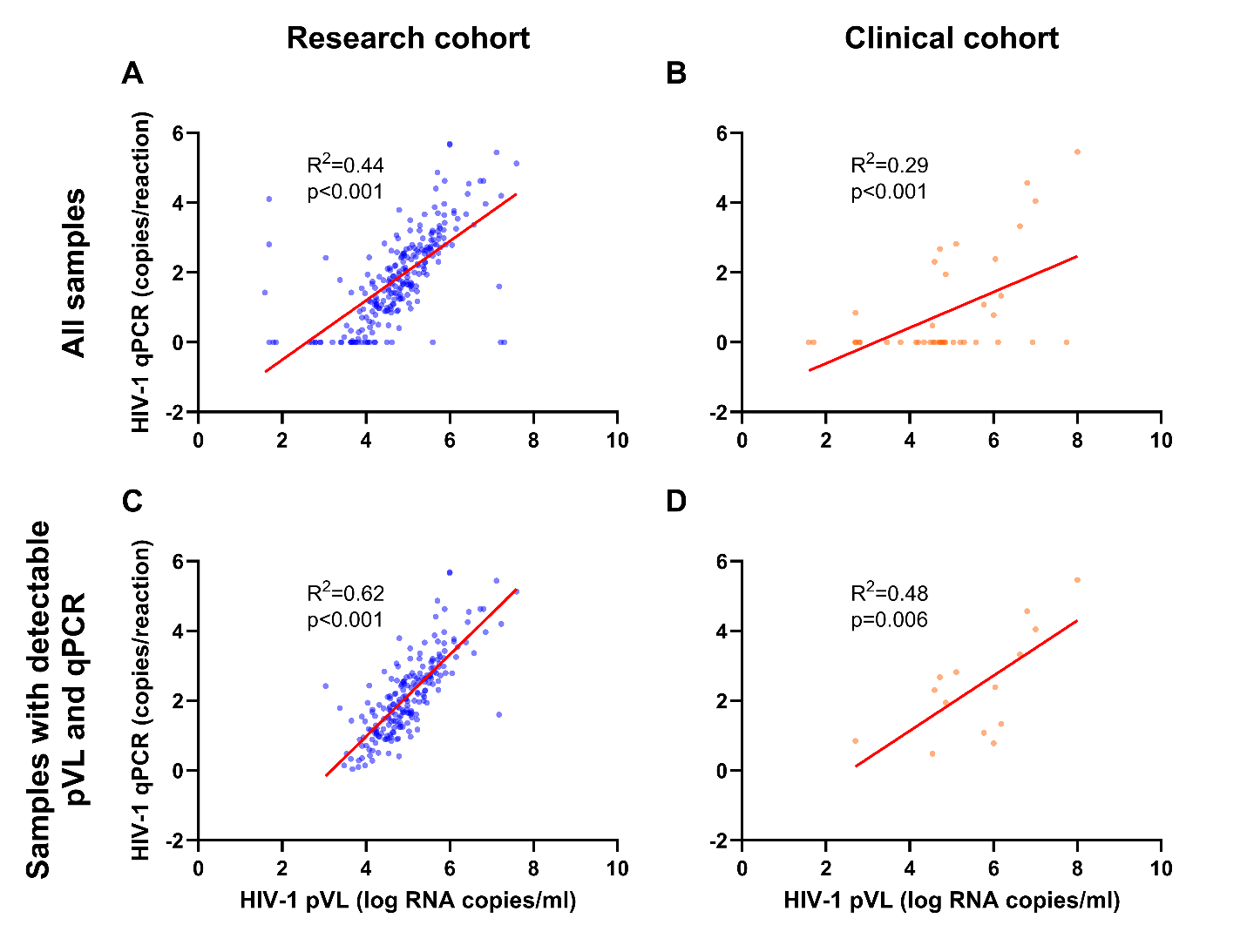


**Figure S3.** **Associations between measurements of HIV-1 RNA at visit (HIV-1 pVL) and during sequencing (HIV-1 qPCR).** All samples in the (A) research and (B) clinical cohorts are shown. A subset of samples with detectable RNA both at visit and during sequencing in (C) research and (D) clinical cohorts are shown. The red line indicates line of best fit, and Pearson’s R^2^ and p-values are shown. Abbreviations: pVL (plasma viral load), qPCR (quantitative polymerase chain reaction).

**
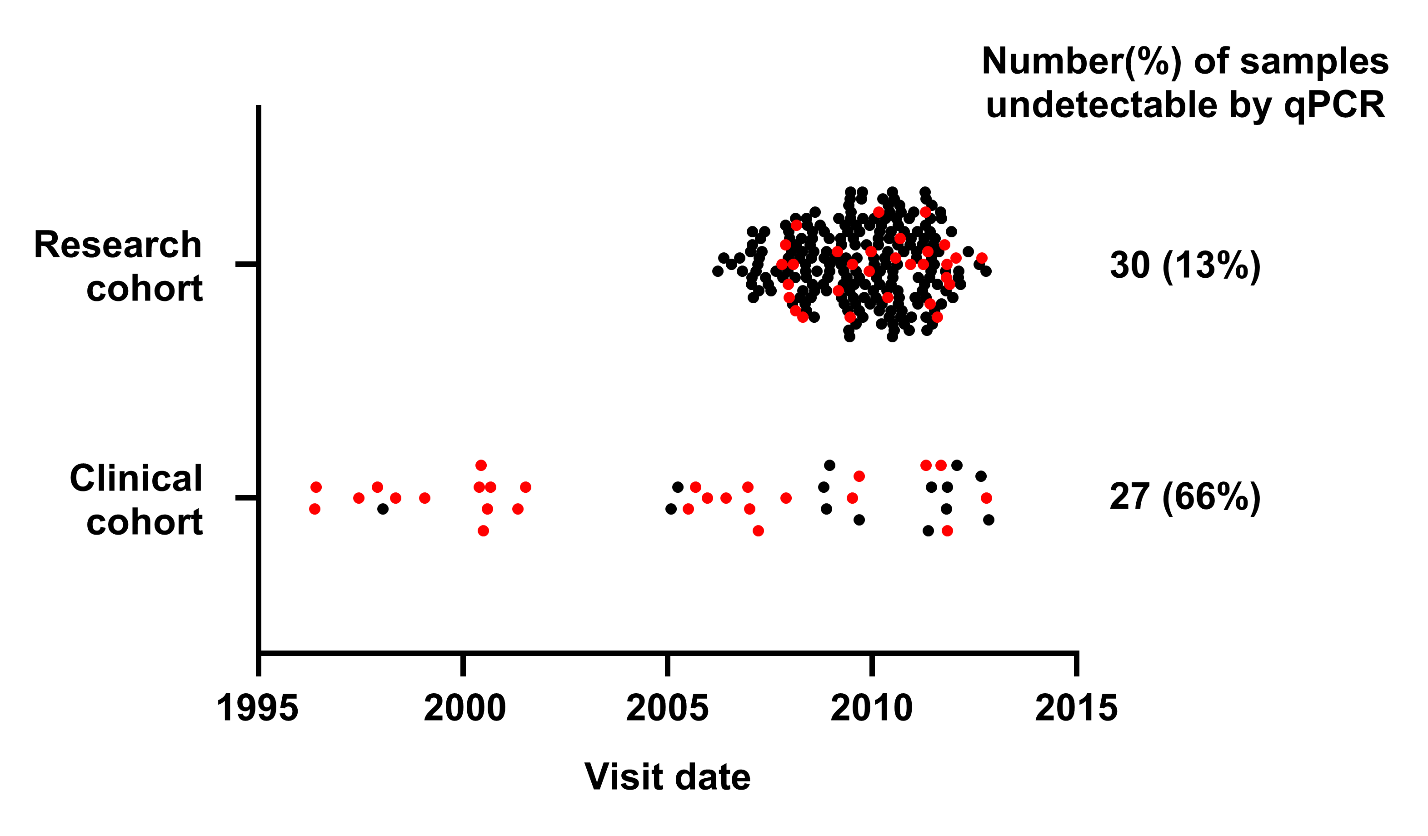
**

**Figure S4. Visit dates of participants in the research and clinical cohorts.** Red dots indicate detectable HIV-1 pVL at visit but undetectable HIV RNA by qPCR during sequencing. Abbreviations: pVL (plasma viral load), qPCR (quantitative polymerase chain reaction).


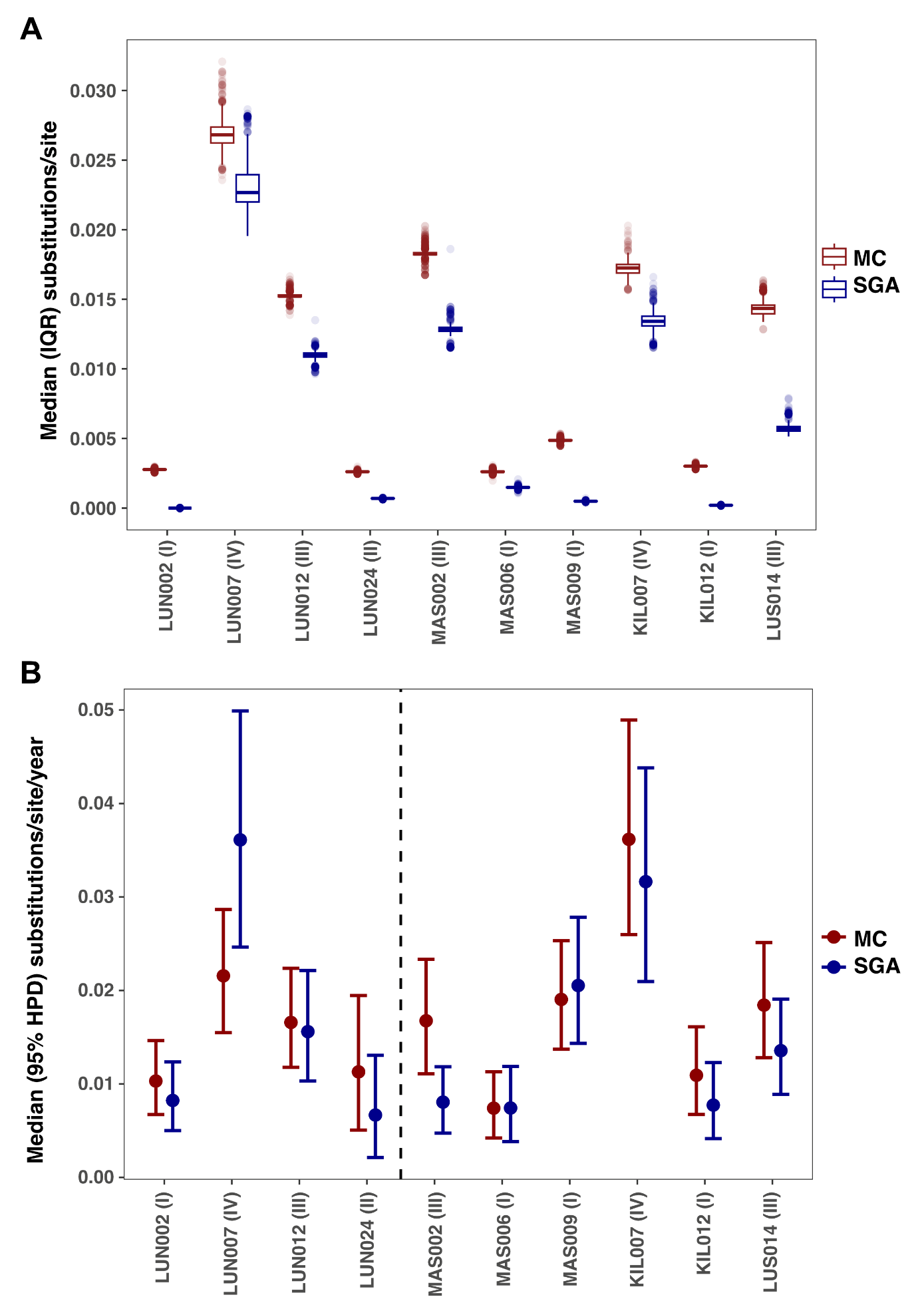


**Figure S5. Diversity and evolutionary rate estimates across sequencing methods when including putatively recombinants.** Recombinant sequences were detected using the PHI test and RDP5 as described in the main text of the article. (A) The average pairwise distance was calculated from maximum-likelihood phylogenetic trees. (B) The evolutionary rates were estimated using BEAST X. Roman numerals in parentheses indicate the time point used. Dotted line separates clinical (left) from research (right) cohort participants. Abbreviations: PHI (Pairwise homoplasy index), BEAST (Bayesian evolutionary analysis sampling trees), MC (molecular cloning), RDP (recombination detection program), SGA (single genome amplification).

**
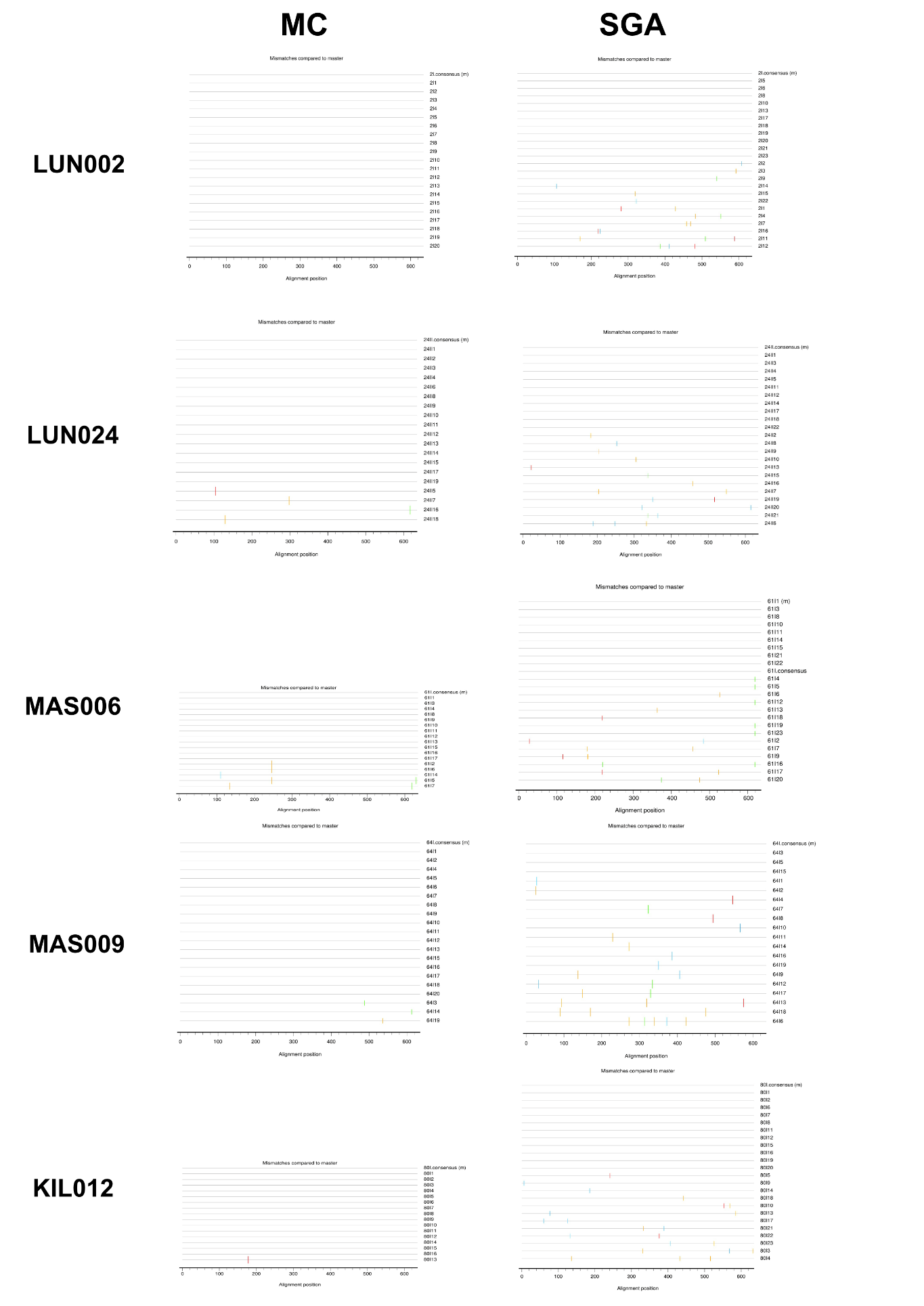
**

**Figure S6. Highlighter plots characterising monophyletic lineages in each of the five participants eligible for transmitted founder virus quantification using either MC or SGA sequences.** Abbreviations: MC (molecular cloning), SGA (single genome amplification).

**Figure S7. Distributions of Hamming distances characterising monophyletic lineages in each of the five participants eligible for transmitted founder virus quantification using either MC or SGA sequences.** Abbreviations: MC (molecular cloning), SGA (single genome amplification).


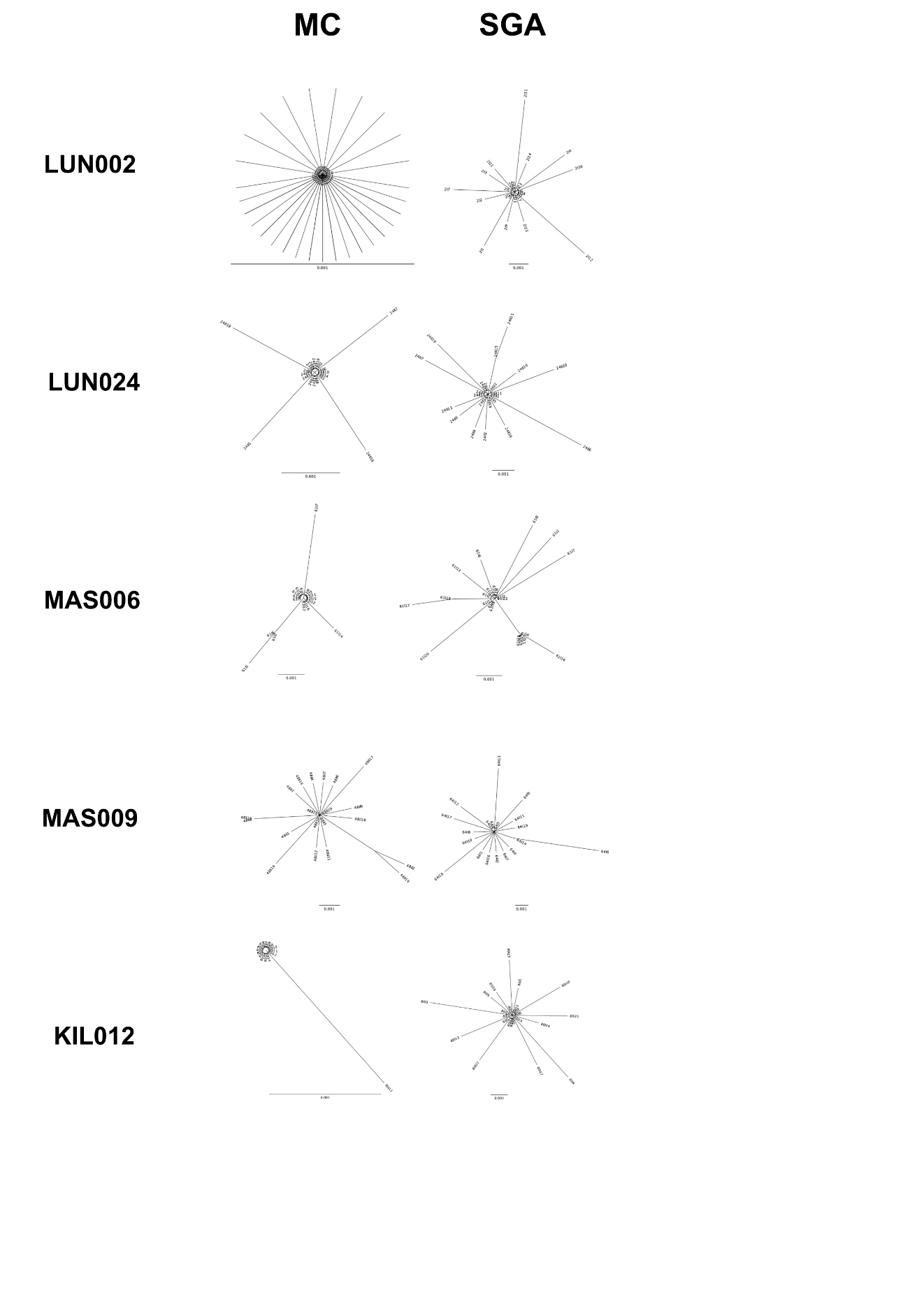


**Figure S8. Neighbour-joining phylogenetic trees characterising monophyletic lineages in each of the five participants eligible for transmitted founder virus quantification using either MC or SGA sequences.** The scale bar represents 0.001 substitutions/site in all trees. Abbreviations: MC (molecular cloning), SGA (single genome amplification).

**Table S1. Root height of tree per participant and method.**

|  |  | **Root height (95% HPD interval)** | |
| --- | --- | --- | --- |
| **Participant ID** | **Time point** | **MC** | **SGA** |
| LUN002 | I | 638.2 [563.8, 758.0] | 607.5 [534.1, 729.4] |
| LUN007 | IV | 553.7 [519.6, 606.4] | 537.5 [509.9, 578.9] |
| LUN012 | III | 735.1 [625.6, 869.1] | 810.5 [664.0, 1008.7] |
| LUN024 | II | 616.9 [541.2, 740.9] | 604.4 [531.4, 731.7] |
| MAS002 | III | 778.6 [637.4, 955.4] | 885.3 [678.5, 1158.0] |
| MAS006 | I | 654.7 [560.9, 793.3] | 605.0 [531.1, 721.2] |
| MAS009 | I | 599.5 [543.3, 681.0] | 546.5 [515.7, 594.1] |
| KIL007 | IV | 734.2 [627.9, 872.6] | 719.3 [617.8, 847.9] |
| KIL012 | I | 632.1 [558.6, 744.6] | 584.9 [526.2, 685.2] |
| LUS014 | III | 653.8 [563.1, 769.2] | 652.5 [555.3, 779.6] |

Abbreviations: ID (identifier), HPD (highest posterior density), MC (molecular cloning), SGA (single genome amplification).

**Table S2. Subtypes per participant.**

| **Participant ID** | **Cohort** | **Subtype** |
| --- | --- | --- |
| LUN002 | Clinical | B |
| LUN007 | Clinical | A1 |
| LUN012 | Clinical | F1 |
| LUN024 | Clinical | B |
| MAS002 | Research | A1 |
| MAS006 | Research | A1 |
| MAS009 | Research | A1 |
| KIL007 | Research | A1 |
| KIL012 | Research | A1 |
| LUS014 | Research | C |

Abbreviations: ID (identifier).
